# Chatbots’ Role in Generating Single Best Answer Questions for Undergraduate Medical Student Assessment: Comparative Analysis

**DOI:** 10.2196/69521

**Published:** 2025-05-30

**Authors:** Enjy Abouzeid, Rita Wassef, Ayesha Jawwad, Patricia Harris

**Affiliations:** 1 School of Medicine, University of Ulster, Northland Road, Derry-Londonderry, BT48 7JL, United Kingdom, 44 7516989748

**Keywords:** artificial intelligence, assessment, Bing, ChatGPT, Gemini, medical education, single best answer

## Abstract

**Background:**

Programmatic assessment supports flexible learning and individual progression but challenges educators to develop frequent assessments reflecting different competencies. The continuous creation of large volumes of assessment items, in a consistent format and comparatively restricted time, is laborious. The application of technological innovations, including artificial intelligence (AI), has been tried to address this challenge. A major concern raised is the validity of the information produced by AI tools, and if not properly verified, it can produce inaccurate and therefore inappropriate assessments.

**Objective:**

This study was designed to examine the content validity and consistency of different AI chatbots in creating single best answer (SBA) questions, a refined format of multiple choice questions better suited to assess higher levels of knowledge, for undergraduate medical students.

**Methods:**

This study followed 3 steps. First, 3 researchers used a unified prompt script to generate 10 SBA questions across 4 chatbot platforms. Second, assessors evaluated the chatbot outputs for consistency by identifying similarities and differences between users and across chatbots. With 3 assessors and 10 learning objectives, the maximum possible score for any individual chatbot was 30. Third, 7 assessors internally moderated the questions using a rating scale developed by the research team to evaluate scientific accuracy and educational quality.

**Results:**

In response to the prompts, all chatbots generated 10 questions each, except Bing, which failed to respond to 1 prompt. ChatGPT-4 exhibited the highest variation in question generation but did not fully satisfy the “cover test.” Gemini performed well across most evaluation criteria, except for item balance, and relied heavily on the vignette for answers but showed a preference for one answer option. Bing scored low in most evaluation areas but generated appropriately structured lead-in questions. SBA questions from GPT-3.5, Gemini, and ChatGPT-4 had similar Item Content Validity Index and Scale Level Content Validity Index values, while the Krippendorff alpha coefficient was low (0.016). Bing performed poorly in content clarity, overall validity, and item construction accuracy. A 2-way ANOVA without replication revealed statistically significant differences among chatbots and domains (*P*<.05). However, the Tukey-Kramer HSD (honestly significant difference) post hoc test showed no significant pairwise differences between individual chatbots, as all comparisons had *P* values >.05 and overlapping CIs.

**Conclusions:**

AI chatbots can aid the production of questions aligned with learning objectives, and individual chatbots have their own strengths and weaknesses. Nevertheless, all require expert evaluation to ensure their suitability for use. Using AI to generate SBA prompts us to reconsider Bloom’s taxonomy of the cognitive domain, which traditionally positions creation as the highest level of cognition.

## Introduction

Across disciplines of education, including medical education, programmatic assessment offers flexible learning modalities that pave the road for individual progression. However, it represents a challenge to educators, as they are required to develop frequent assessments that reflect different competencies, thus necessitating the continuous creation of examination content in a comparatively restricted time [1]. For many years, multiple choice questions (MCQs) have been adopted in medical education for assessing knowledge and clinical reasoning skills in high-stakes undergraduate and postgraduate medical exams. MCQs are reliable, objective, standardized, equitable, and efficient formats for testing large volumes of content in a limited time. A main problem with MCQs is that producing high-quality questions is time-consuming, from drafting the question that includes a clinical vignette or stem, a lead-in question, a correct answer, and distractors to validation of content and detection of potential flaws [1,2]. To tackle this dilemma, the application of many technological innovations, including artificial intelligence (AI), has been tried [3].

AI refers to machines mimicking the human brain in performing intellectual tasks. This originates from the imitation game developed by the British mathematician Alan Turing, who posed the universally famous question “Can machines think?” [4]. Since then, many AI research laboratories have invested time, effort, and money to answer this question. One particular AI research laboratory known as OpenAI, based in California, United States, has revolutionized our world at the end of 2022 by launching an AI-based large language model (LLM) software (GPT-3.5) that uses natural language processing to engage in human-like conversations and making it freely available for the public [5]. Within a few weeks after its release, the OpenAI chatbot, known as ChatGPT, had gained much attention in many fields, including medical education. It became the fastest-growing app of all time with more than 120 million users in just a few months after its launch [6]. This led competitors to develop and launch other chatbots. Microsoft launched Bing Chat AI in February 2023, followed by Google releasing Gemini in March 2023 [7]. A newer, improved version of ChatGPT (ChatGPT Plus), which uses the GPT-4 Turbo language model, has been developed by OpenAI and launched as a paid subscription version by the end of 2023 [6]

In terms of assessment in medical education, ChatGPT has been the most extensively studied chatbot. It was found to be able to quickly and accurately apply known concepts in medicine to novel problems, including reflection prompts and examination questions, and to mimic human writing styles, introducing a potential threat to the validity of traditional forms of medical student assessment including short answer assessment [8], it even successfully passed the USMLE (United States Medical Licensing Examination) [9]. Similarly, ChatGPT-4 was able to achieve a mean of more than 75% in the newly derived undergraduate medical exit examination: UKMLA (United Kingdom Medical Licensing Assessment) [10]. Its application has been described across multiple areas of academic assessment, for example, developing innovative assessments, grading submitted work, and providing feedback [11]. Nevertheless, concerns persist around the validity of the information provided by all AI tools. Sample [12] argued that if the chatbot response is not properly verified, it can be misleading and result in “junk science.”

Additionally, the broad availability of LLMs such as ChatGPT, Gemini, and Bing has facilitated extensive comparative studies across various domains. For example, 1 study evaluated these models using case vignettes in physiology and found that ChatGPT-3.5 outperformed Bing and Google Bard (an old version of Gemini), indicating its superior effectiveness in case-based learning [13]. Another study, using the clinicopathological conferences method, compared the ability of AI chatbots to infer neuropathological diagnoses from clinical summaries. The findings revealed that Google Bard and ChatGPT-3.5 correctly diagnosed 76% of cases, while ChatGPT-4 achieved a higher accuracy rate, correctly identifying 84% of cases [14]. Similarly, a comparison of ChatGPT-3.5, Google Bard, and Microsoft Bing in hematology cases highlighted significant performance differences, with ChatGPT achieving the highest accuracy [15].

Recent studies have explored the use of AI in generating MCQs and single best answer (SBA) questions for medical examinations, highlighting its potential applications and limitations. For instance, Zuckerman et al [16] examined ChatGPT’s role in assessment writing, while Kıyak et al [17] and Mistry et al [18] investigated AI-generated MCQs in pharmacotherapy and radiology board exams, respectively.

Despite these contributions, the ability of AI to generate valid SBA questions, an assessment format that better evaluates higher-order cognitive skills such as data interpretation, problem-solving, and decision-making [19], remains an area requiring further exploration. Additionally, a critical consideration is the variation in AI-generated outputs and the potential for examination candidates to predict examination items based on curriculum learning objectives (LOBs). Given the significance of these issues, this study aims to examine the content validity and consistency of different chatbots in generating SBAs for undergraduate medical education.

## Methods

### Study Context

The Graduate Entry Medical Programme at Ulster University’s School of Medicine is a 4-year program. Similar to most UK medical schools, students undergo assessment through a series of SBA papers comprising over 1500 questions across the program. Managing this extensive assessment requirement has prompted the exploration of innovative solutions to support the assessment team.

To ensure assessment standards, the school has implemented a rigorous quality assurance process. Questions are first created by designated clinical or academic authors who have been trained and provided with a “house style” to follow. Questions then undergo internal review by other clinical or academic staff before external review by external examiners to ensure they meet rigorous requirements. Post hoc psychometric analysis of question performance is also used to drive evidence-based review and enhancement. This meticulous review process aims to uphold the integrity and effectiveness of assessments used to make high-stakes progression decisions and forms part of a wider suite of quality processes to deliver against the assessment strategy.

### Study Design

**Figure 1.**
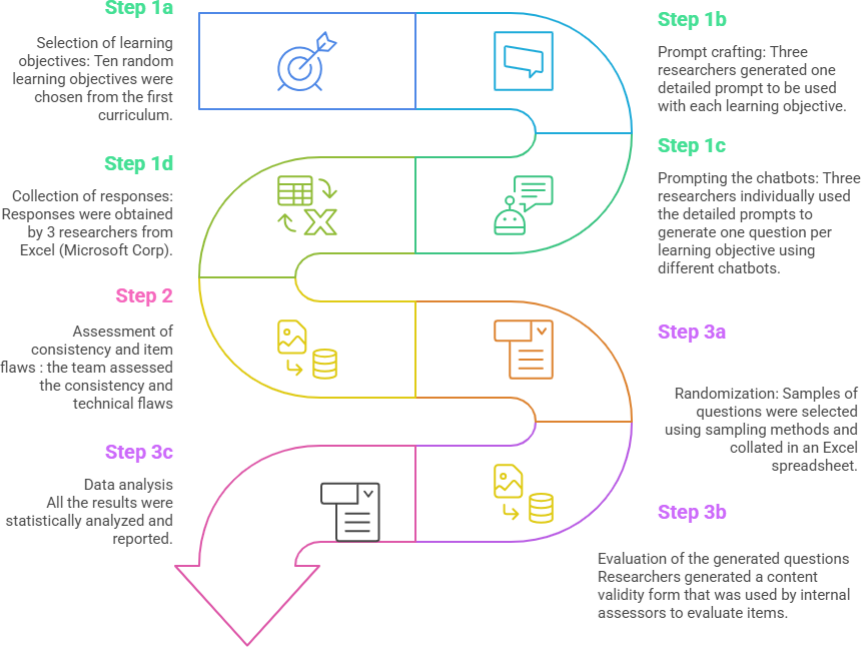
The study design. SBA: single best answer.

This exploratory comparative study was conducted between December 2023 and May 2024; we continued to follow the school’s established quality assurance process, but the designated first authors of the questions were AI chatbots. This includes 3 versions of AI chatbots: ChatGPT which will be referred to as ChatGPT-3.5 in this study, Google Gemini, and Microsoft Bing AI, in addition to the subscription-only version of OpenAI: ChatGPT-4 that provides access to GPT-4 Turbo, which is advertised as a more powerful and faster version of GPT-4. During this study, Google changed the name of its platform from Bard to Gemini. For consistency, this paper will refer to the current name: Gemini. Figure 1 depicts the full study design, which included three main phases: (1) Generation of questions using various AI chatbots, (2) Assessment of the consistency of the chatbot outputs, and (3) Evaluation of the quality of the questions generated.

### Generation of Questions Using Various AI Chatbots

In phase one, the research team randomly selected year 1 curriculum LOBs (n=10) to create SBA questions for. These objectives were selected using stratified random sampling from the official list of LOBs for second-semester educational units. Three researchers were involved, and each one created a new account for each of the 4 chatbot platforms. All researchers used the same predefined prompts (see below) around the same time (end of December 2023) to request 10 questions from each chatbot, one for each LOB. The 10 prompts were entered one by one in the same conversation with each chatbot. All the questions were compiled into a shared Microsoft Excel (Microsoft Corp) spreadsheet for analysis in steps 2 and 3.

To allow a fair comparison, the same prompt was used in each chatbot, which specified SBA features:

- You are a university lecturer in a UK medical school. Generate an MCQ on “the learning objective,” with the following criteria:
  - The question is in a clinical vignette format.
  - The question is designed to assess the knowledge (±clinical judgment) of undergraduate medical students.
  - The question meets the standard for a medical graduate examination.
  - Five choices are allowed for each question.
  - Only 1 correct answer
  - Tag the correct answer.
  - Justify the correct answer.

### Assessment of the Consistency and Quality (Item Flaws) of the Chatbot Outputs

In the second phase, researchers involved in the previous step assessed each chatbot’s output consistency and technical flaws. Consistency was evaluated based on the similarity between the outputs generated across the 3 researchers, including any bias in the correct answer allocation (eg, favoring option “A” as the correct answer). Similarity was evaluated based on specific elements of the output and accordingly classified into one of three categories: (1) exact questions: when the outputs contain the same wording, condition, and lead-in question; (2) similar questions: when the outputs share common elements such as patient characteristics, age, condition, presentation, or lead-in question; (3) different questions: when the outputs do not have any content in common.

Technical item flaws assessed the overall construct and structure of the questions produced by the chatbots using 7 previously published criteria for determining the quality of SBAs [20]. The 7 criteria include judgments on whether the questions: follow the SBA structural format, satisfy the “cover test” rule where the question should be answerable solely from the vignette or stem and lead-in (with the answers “covered”), test the application of knowledge rather than recall isolated facts, have item balance (which ensures a balance in information between the stem, lead-in, and options), tests 1 idea, are dependent on the vignette to reach the correct answer, and have appropriate lead-ins length. The researchers used a defined scale to evaluate how often or to what extent each criterion was met across the 3 researchers’ outputs. Each criterion was scored on a scale from 0 to 3 for each of the 10 LOB prompts. In this scale, 0 meant none, 1 meant 1 SBA, 2 meant 2 SBAs, and 3 meant all 3 SBAs, representing the number of questions produced by each chatbot that met the criterion. With 3 assessors and 10 LOBs, the maximum possible score for any individual chatbot was 30.

### Assessment of the Content Validity and Accuracy of the Questions Generated

In phase 3, samples of questions generated by the chatbots were distributed to various internal assessors as per our normal quality review process. The questions were selected using stratified random sampling to select 1 of the 3 questions generated by each chatbot for each LOB, yielding a total of 39 questions. Alongside this, a content validation evaluation form, developed by the research team, was used to ensure consistent review between assessors, providing assessors with clear expectations and an understanding of the task. The assessors are faculty members with expertise in the curriculum content. Each question was evaluated by 7 assessors.

Considering published recommendations for content validation [21,22], 20 internal assessors were invited, of which 7 consented to participate. The internal assessors critically reviewed the questions based on several criteria to ensure their quality and alignment with educational objectives. This includes content clarity and validity; accuracy of information, answers, and justification; and educational accuracy. Each of these elements was scored on a Likert scale of 1 to 4 (with 1 representing the lowest level of construct and 4 the highest level of the construct; Multimedia Appendix 1).

### Statistical Analysis

Quantitative data was analyzed through scores obtained from the rating scale using IBM SPSS Statistics (version 26; IBM Corp). Subsequently, 2 content validity indexes were computed: the Item Content Validity Index (I-CVI) and the Scale Level Content Validity Index (S-CVI). Percentages and frequencies were calculated for the questions’ scores to provide further insights into the data. A 2-way ANOVA without replication was conducted to assess differences in chatbot performance across 6 domains. Post hoc comparisons were performed using the Tukey-Kramer HSD (honestly significant difference) test to identify specific group differences. The average ratings provided by 7 evaluators were used for each chatbot and each criterion. The Krippendorff alpha [23] was used to assess interrater reliability, using the K-Alpha Calculator [24]. A coefficient value of 0.8 is considered satisfactory [23]. However, the low Krippendorff alpha suggested a need for further refinement of the rating scheme or additional training for raters to improve reliability.

### Ethical Considerations

Participants were informed that their responses would be anonymized and that they could withdraw from this study at any point without penalty. Informed consent was obtained from all participants before data collection. Only those who provided explicit consent were included in this study. This study received ethical approval from the Ulster University Centre for Higher Education Research and Practice Ethics Committee and the Learning Enhancement Directorate Ethics Filter Committee (LEDEC; formerly CHERP; LEDEC-24-004). All data were anonymized during the analysis phase to ensure confidentiality and to protect participants’ identities. Staff members who chose not to participate experienced no disadvantage or impact on their professional standing. No financial or material compensation was offered to participants for their involvement in this research.

## Results

### Generation of Questions

In response to the predefined prompts provided to the chatbots, 3 of them (free ChatGPT, ChatGPT Plus, and Gemini) generated 10 questions each, for a total of 30 across the 3 researchers. Bing could not respond to the prompt for LOB9 and thus generated 9 questions, for a total of 27 across the 3 researchers. Thus, 117 questions were generated (Multimedia Appendix 2).

### Assessment of Consistency Within Chatbots and Technical Item Flaws Among the Outputs

Consistency within chatbots was evaluated based on the similarity of outputs between the 3 researchers and any bias in the allocation of the correct answer option. Bing had the highest degree of similarity between items generated by multiple users (4 exact question matches and 20 similar ones), while ChatGPT-4 had the highest degree of variation (Table 1).

**Table 1.** Similarity between the questions generated by different chatbots.

	Gemini (N=30), n (%)	Bing (N=27), n (%)	ChatGPT-3.5 (N=30), n (%)	ChatGPT-4 (N=30), n (%)
Exact questions	0 (0)	4 (14.81)	2 (6.67)	0 (0)
Similar questions	24 (80)	20 (74.07)	22 (73.33)	22 (73.33)
Different questions	6 (20)	3 (11.11)	6 (20)	8 (26.67)

The original predefined prompt did not request answer options to be given in any particular order. Therefore, for assessing potential bias in the correct answer allocation, 3 scenarios were modeled (Table 2):

- Any bias or preference in the correct answer allocation based on the raw chatbot output.
- Any bias or preference in the correct answer allocation based on the chatbot output when the researchers manually ordered answers into alphabetical order.
- Any bias or preference in the correct answer allocation based on a new output, where each chatbot was prompted to produce 30 new SBA questions with answers alphabetically.

**Table 2.** Assessment of possible bias or preference in correct answer allocation.

Options		Gemini (N=30), n (%)	Bing (N=27), n (%)	ChatGPT-3.5 (N=30), n (%)	ChatGPT-4 (N=30), n (%)
Original chatbot output					
	A	5 (16.67)	6 (22.22)	9 (30)	11 (36.67)
	B	12 (40)	4 (14.81)	10 (33.33)	10 (33.33)
	C	6 (20)	10 (37.04)	7 (23.33)	4 (13.33)
	D	5 (16.67)	6 (22.22)	3 (10)	4 (13.33)
	E	2 (6.67)	1 (3.7)	1 (3.33)	1 (3.33)
Manual reordering of chatbot output into alphabetical order					
	A	4 (13.33)	8 (29.63)	8 (26.67)	6 (20)
	B	10 (33.33)	3 (11.11)	3 (10)	7 (23.33)
	C	3 (10)	5 (18.52)	7 (23.33)	5 (16.67)
	D	9 (30)	4 (14.81)	6 (20)	5 (16.67)
	E	4 (13.33)	7 (25.93)	6 (20)	7 (23.33)

Gemini, ChatGPT-3.5, and ChatGPT-4 occasionally provided answer options in alphabetical order when not specifically prompted. Gemini consistently demonstrated a preference for the correct answer to be listed as option B. The ChatGPT-3.5 and ChatGPT-4 appeared to favor options A, B, and C. Bing appeared to favor options A and E.

**Figure 2.**
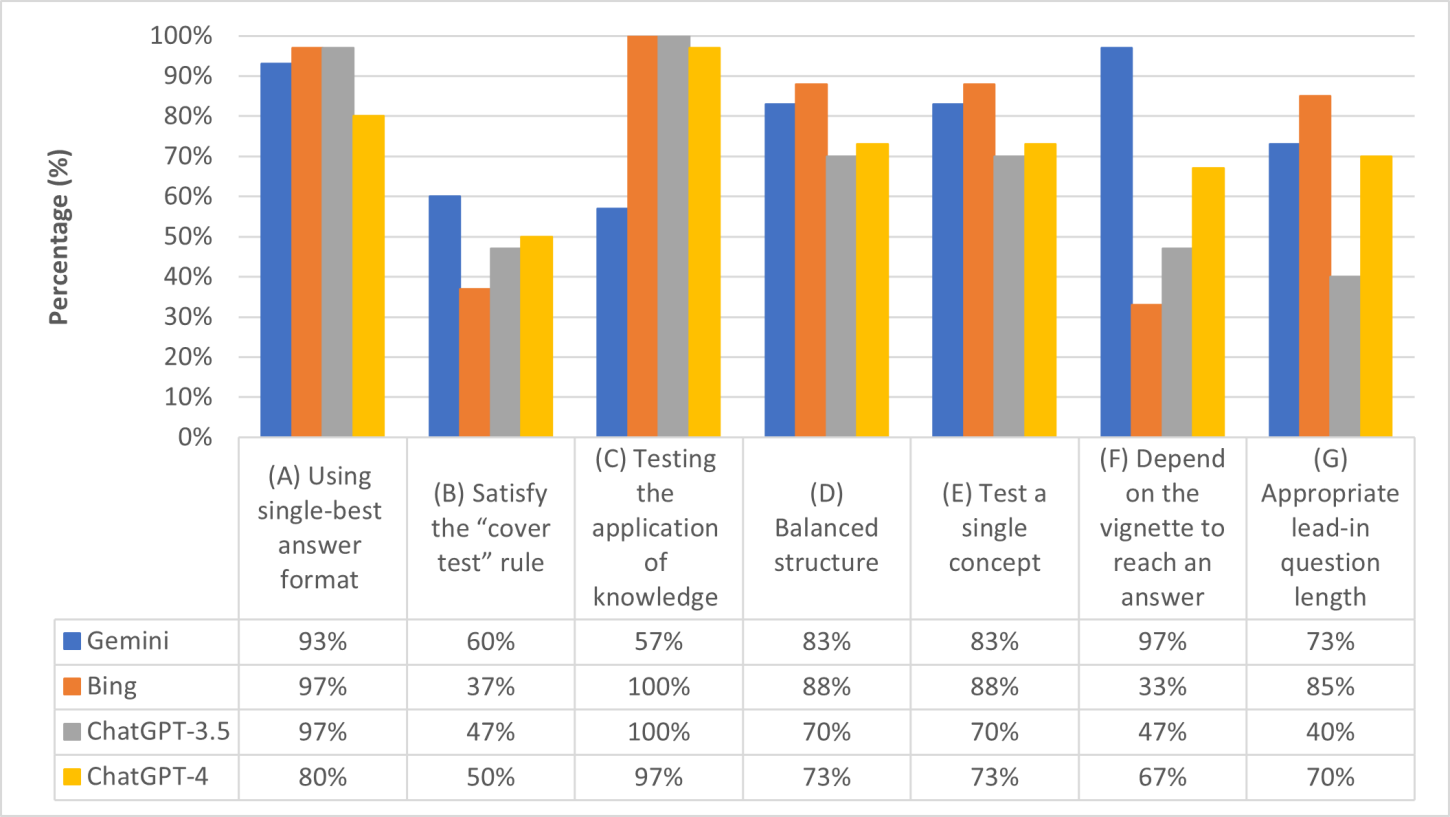
Shows technical item flaws among the chatbots: (**A**) single best answer format, (**B**) satisfy the “cover test” rule, (**C**) test the application of knowledge rather than recall isolated facts, (**D**) questions were balanced, (**E**) lead-in question tests one idea, (**F**) questions depend on the vignette to reach an answer, and (**G**) appropriate lead-in question length. The total number of questions generated by Bing was 27.

Regarding the technical item flaws among the outputs, the chatbots performed similarly in terms of following an SBA format (Figure 2A) and achieving the “cover test” satisfaction (Figure 2B), although ChatGPT-4 scored slightly lower on satisfying the cover test. Overall, Gemini performed well across most items, except for item balance. Notably, Gemini stood out by creating questions with a lead-in that relied heavily on the vignette for the answer (Figure 2F). Bing scored low across most evaluation items but performed well in generating a lead-in question of appropriate length (Figure 2G). ChatGPT Plus, which required a paid subscription, did not outperform the other chatbots in any item. The evaluation item “questions test the application of knowledge rather than recall of isolated facts” received the lowest scores across all the chatbots (Figure 2C), with Gemini achieving the highest score among them.

### Assessment of Content Validity and Accuracy

Seven internal assessors evaluated item clarity and relevance, deriving the I-CVI for individual SBA items and the S-CVI (following the Universal Agreement method) to assess the overall content validity for questions from each chatbot (Table 3). Items with I-CVI>0.79 and scales with S-CVI/UA>0.8 can be interpreted as acceptable [20].

Assessors also evaluated items for content clarity and 4 elements of accuracy: vignette information, answers, justifications, and educational accuracy, on a scale from 1 to 4 (Tables4  5). The Krippendorff alpha coefficient was low, 0.016, with a 95% bootstrap CI of −0.066 to 0.116.

As depicted in Tables3  4, SBA questions from 3 chatbots (ChatGPT, Gemini, and ChatGPT Plus) had similar content clarity and S-CVI values. In comparison to the other chatbots, Bing performed worst in content clarity, overall (scale) validity, and all elements of item accuracy. ChatGPT Plus, which required a paid subscription, did not outperform the other chatbots except in the measure of educational accuracy. Further statistical analysis was performed using the 2-way ANOVA without replication, which showed statistically significant differences among chatbots and domains (*P*<.05). However, the Tukey-Kramer HSD post hoc test revealed no significant pairwise differences between individual chatbots, as all comparisons had *P* values>.05 and overlapping CIs. Thus, although the chatbots’ performance varied overall, specific chatbot differences were not statistically significant.

**Table 3.** Item-content validity and scale-content validity across the chatbots.

Item number	Gemini	Bing	ChatGPT-3.5	ChatGPT-4
I-CVI^a^				
1	1	1	1	1
2	1	1	1	1
3	1	1	1	1
4	1	1	1	1
5	0.85	0.85	0.85	0.83
6	0.85	0.85	0.71	0.85
7	0.85	0.85	0.85	0.85
8	0.85	0.85	0.85	0.85
9	0.85	—^b^	0.85	0.85
10	0.85	0.85	0.85	0.85
S-CVI/UA^c^	0.91	0.83	0.9	0.91

a I-CVI: Item Content Validity Index.

b Not applicable.

c S-CVI/UA: Scale Level Content Validity Index.

**Table 4.** Average score for content clarity and accuracy of items across the chatbots.

	Content clarity^a^	Accuracy of information^b^	Accuracy of answers^c^	Accuracy of justification^d^	Educational accuracy^e^
Gemini	3.68	3.71	3.8	3.91	3.49
Bing	3.41	3.3	3.49	3.47	3.2
ChatGPT-3.5	3.75	3.71	3.84	3.9	3.5
ChatGPT-4	3.71	3.66	3.81	3.82	3.56

a Content clarity refers to the extent to which the question is clearly written, free of ambiguity, and easily understood by the intended audience.

b Accuracy of information verifies that the facts, concepts, and explanations presented are scientifically and contextually correct.

c Accuracy of answers ensures that the correct response is indeed accurate, while the distractors remain plausible yet distinguishable.

d Accuracy of justification evaluates whether the rationale provided for correct and incorrect answers is logically sound, evidence-based, and supports a deeper understanding of the topic.

e Educational accuracy assesses whether the question is appropriately challenging to the student level, measures higher cognitive levels (such as application or analysis), and adheres to best practices in assessment design.

**Table 5.** Two-way ANOVA table.

Source of variation	Sum of squares due to the source	*df*	Mean sum of squares due to the source	*F* test	*P* value
Average content clarity and accuracy scores	0.304357	2	0.152178	24.26587	<.001
Chatbots	17.9744	4	4.493601	716.5349	<.001
Error	0.05017	8	0.006271	—^a^	—
Total	18.32893	14	—	—	—

a Not applicable.

## Discussion

### Interpretation of Findings

This study was designed to examine the content validity and consistency of SBA questions generated by different chatbots in the context of undergraduate medical education. The findings revealed that no single chatbot excelled in all studied domains nor demonstrated a universal superiority over other chatbots, but rather showed unique strengths of some chatbots in specific areas and highlighted their notable limitations in other ones. This emphasizes the importance of critically assessing the output of chatbots in a context-sensitive manner. Bing produced items that were least suitable for inclusion in medical student assessment. These findings echo previous studies, which also show Bing to generate less valid MCQs in comparison to other chatbots [25]. ChatGPT-4 showed the greatest variation in responses across users (suggesting higher protection against examination candidates predicting potential assessment items), and had strong performance in content clarity and accuracy, though it also exhibited some less effective question design practices, such as poorer performance in the “cover test” rule. These findings align with the results of Doughty et al [26], who found that GPT-4’s ability to generate effective MCQs was nearly on par with human performance, in which 81.7% of the generated MCQs met all evaluation criteria, suggesting that fewer than 1 in 5 questions would need revision by instructors. However, in cases where ChatGPT-4 failed to meet a quality standard, this was typically the only issue with the question. Gemini performed well across all evaluations, matching ChatGPT Plus’s strong index score for content validity, and excelled in creating questions where the lead-in tested 1 item and relied heavily on the vignette for the answer. Although slightly behind both ChatGPT versions in content clarity, Gemini scored the highest in providing accurate justifications for the correct answer.

This variation across chatbots is consistent with results from studies where chatbots were asked to answer questions. Kumari et al [15] found significant differences in solving hematology case vignettes using LLMs. ChatGPT achieved the highest score, followed by Google Gemini and then Microsoft Bing. In line with this, Dhanvijay et al [13] reported that ChatGPT-3.5 scored the highest, Bing the lowest, and Bard (Gemini) ranked in the middle when solving case vignettes in physiology. When chatbots were tested on their ability to answer SBA questions, ChatGPT-4 and Microsoft Copilot (Bing) outperformed Google Gemini [27]. Overall, these results suggest that OpenAI’s ChatGPT shows strong potential in the medical education field. However, it is worth noting that none of the models were able to answer all questions correctly, and in our study, all platforms had some flaws when generating SBAs.

Additionally, this study’s results reveal several key insights and revelations concerning SBA questions produced by AI chatbots. First, we observed that chatbots often exhibit a correct answer bias toward particular options. Recent studies have identified that LLMs tend to display positional bias when handling MCQs [28,29]. Radford et al [30] and Li and Gao [31] found that this susceptibility to positional bias is pronounced in the GPT-2 family however a more recent technical report for GPT-4 suggests AI’s performance in MCQ remains susceptible to the position of the correct answer among the choices [32], a pattern referred to as “anchored bias.” To minimize this inherent bias that appears to occur across AI platforms, when using AI to generate MCQ or SBA, we would recommend not stipulating an order for answer options in the prompt.

Furthermore, assessment literature emphasizes that high-quality SBA questions should assess the higher levels of Bloom’s taxonomy to encourage students’ critical thinking and complex problem-solving [33]. Our study revealed that chatbots were not always successful in crafting questions that engaged these advanced cognitive levels, and this was an area of relative weakness when evaluating items. Gemini scored highest, followed by ChatGPT Plus, ChatGPT-3.5, and then Bing. Similar findings regarding ChatGPT’s limitations were reported by Herrmann-Werner et al [34]. Likewise, studies by Klang et al [35] and Liu et al [36] also emphasized GPT-4’s limited ability to integrate knowledge and apply clinical reasoning, highlighting challenges in logical reasoning, which could limit AI’s ability to generate questions that test this concept. However, it should be noted that while human-written questions were rated higher in direct comparisons, the score gap was narrow and largely insignificant, suggesting that AI tools still hold potential as educational aids [2].

Our analysis also revealed some technical flaws, variations, and inconsistencies in item construction within all chatbots. These flaws highlight instances of overconfidence and inadequacies in question design, suggesting an inability of the chatbots to evaluate their output’s consistency, relevance, and complexity. Flawed MCQs hinder the accurate and meaningful interpretation of test scores and negatively impact student pass rates. Therefore, identifying and addressing technical flaws in MCQs can enhance their quality and reliability [37]. Similarly, Klang et al [35] reported that approximately 15% of questions generated using detailed prompts required corrections, primarily due to content inaccuracies or methodological shortcomings. These revisions often involved addressing a lack of sensitivity in certain topics, such as failing to include specific details such age, gender, or geographical context in the questions or answers.

Most of the questions tested recall and comprehension levels, but Gemini included some that assessed the application of knowledge. In contrast, Bing struggled to generate questions on specific topics. These findings can be explained as critical thinking at higher levels involves considering evidence, context, conceptualization, methods, and the criteria required for judgment [38]. AI models are trained on large datasets of text, but they may not fully understand the context or underlying concepts behind the content. Higher-order thinking skills, such as application, analysis, and synthesis, require deeper comprehension and reasoning that AI might not be able to simulate effectively.

Thus, using AI to generate SBAs encourages us to reconsider Bloom’s taxonomy of the cognitive domains [39,40], which traditionally positions “creation” as the highest level of cognition. In the era of AI, evaluation might be considered the most critical level of cognition [41]. While AI chatbots can often produce well-written questions aligned with LOBs, they still require expert evaluation to ensure their suitability for use. Future research should compare AI-generated outputs with those from subject matter experts to assess accuracy and relevance. Evaluating AI’s ability to test higher-order cognition in Bloom’s taxonomy is also crucial. As AI evolves, ongoing validation is essential to ensure reliability and effectiveness in assessments.

Despite the methodological rigor and innovative approach of this study, some limitations need to be highlighted to improve the interpretation of the findings presented here. First, the researchers or assessors generated or evaluated only 30 questions per chatbot. Variation was observed in the content validity and accuracy between the SBAs produced by an individual chatbot. Therefore, this sample may not sufficiently represent the wide range of possible outputs, potentially limiting the generalizability and robustness of the findings. Second, the accuracy of the chatbots’ responses may have been compromised by the absence of reference materials, which could have negatively affected their performance. Finally, this study is limited by low interrater reliability and the use of measures are not specifically designed to assess MCQ quality. Future research should consider using validated tools to enhance evaluation accuracy.

### Conclusions

Chatbot platforms varied in their ability to generate educational questions. ChatGPT models produced the most variable outputs, reducing predictability while maintaining strong content clarity and accuracy with minimal answer bias. Gemini performed similarly but showed a strong preference for 1 option, while Bing had the least variation and the lowest content clarity and accuracy. ChatGPT-4 did not significantly improve question quality but maximized variability. Technical flaws were present across all platforms, with many questions poorly linked to vignettes. Most tested recall and comprehension, though Gemini included some application-level items, whereas Bing struggled with specific topics.

These findings highlight AI’s limitations in generating higher-order thinking questions, reinforcing the need for expert evaluation. This challenges Bloom’s taxonomy’s traditional cognitive hierarchy, suggesting that “evaluation” may be more critical than “creation” in AI-assisted assessments.

## Supplementary material

10.2196/69521 Multimedia Appendix 1 Further data on the assessment of questions generated

10.2196/69521 Multimedia Appendix 2 Questions generated.

## Abbreviations

AI: artificial intelligence
HSD: honestly significant difference
I-CVI: Item Content Validity Index
LLM: large language model
LOB: learning objective
MCQ: multiple choice question
S-CVI: Scale Level Content Validity Index
SBA: single best answer
UKMLA: United Kingdom Medical Licensing Assessment
USMLE: United States Medical Licensing Examination
